# P-942. Impact of Interdisciplinary Antibiotic Time Out Integrated into Medical Education

**DOI:** 10.1093/ofid/ofaf695.1145

**Published:** 2026-01-11

**Authors:** Michael J Fox, Erika J Monacelli, Philip J Scavo, Jeremy J Agostinho

**Affiliations:** Geisinger Community Medical Center, Bear Creek Twp, PA; Geisinger Community Medical Center, Bear Creek Twp, PA; Geisinger Community Medical Center, Bear Creek Twp, PA; Geisinger Community Medical Center, Bear Creek Twp, PA

## Abstract

**Background:**

Antimicrobial Stewardship Programs are an essential component of acute care practice and key components are recommended by the Center for Disease Control and the Joint Commission. Recommendations include use of an Antibiotic Time Out as a component of stewardship to perform prospective audit and feedback of antimicrobial therapy.

**Figure:**
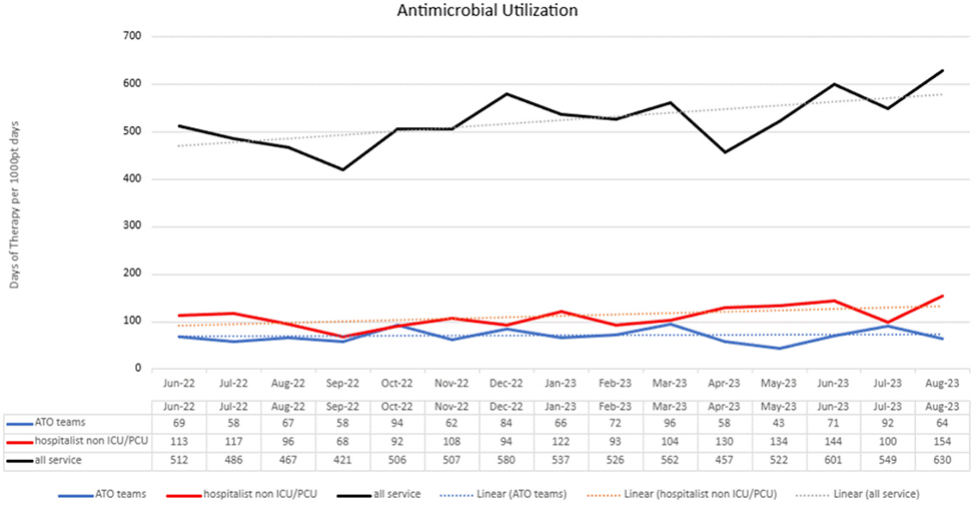

**Figure:**
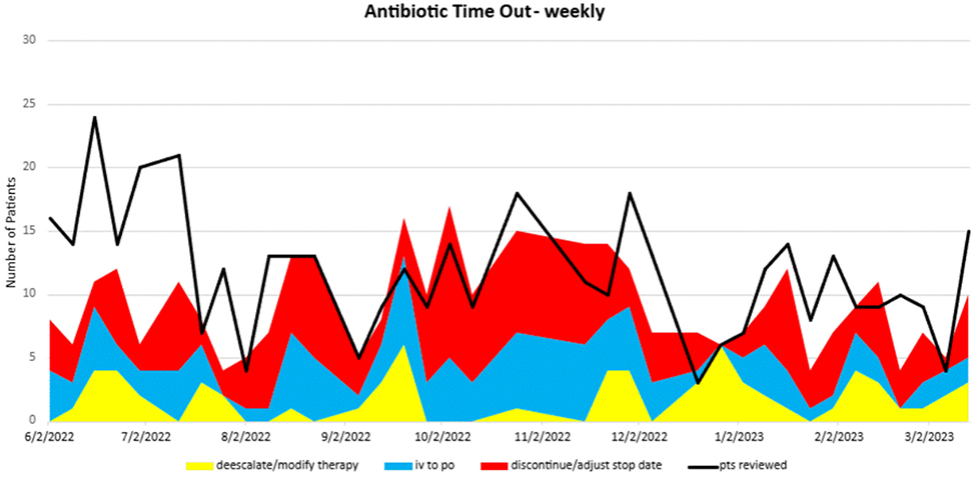

**Methods:**

This was a prospective observational study at an academic medical center evaluating the impact of inclusion of an antibiotic stewardship time out as part of medical education regarding the utilization of antimicrobials. Non-teaching hospitalist services were evaluated against teaching hospitalist services matched by location and acuity. Services covering critical care, progressive care, surgery services, and emergency medicine were excluded from the evaluation. Antibiotic time outs consisted of a weekly review of all patients receiving antimicrobials on the teaching hospitalist services. Senior residents presented to an interdisciplinary stewardship team led by an infectious disease physician and pharmacist where therapy was evaluated, discussed, education performed, and recommendations were made.

**Results:**

From June of 2022 through August 2023, 418 individual patients were reviewed during antibiotic time out resulting in 327 interventions made to antimicrobial therapy (66 de-escalate/modify therapy, 104 intravenous to oral changes, 160 discontinue/adjust stop date) with an intervention rate of 85.49%. Evaluation of the antimicrobial utilization between teaching service teams and non-teaching service hospitalists showed a decrease in antimicrobial consumption measured in days of therapy per 1000 patient days (p< 0.001).

**Conclusion:**

Implementation of an interdisciplinary antimicrobial time out as a component of medical education shows an association with a decrease in antimicrobial utilization as compared to matched non-resident hospitalist services. The impact of this intervention resulted in expanding the program to additional services and other system campuses.

**Disclosures:**

All Authors: No reported disclosures

